# Impairment of emotional expression detection after unilateral medial temporal structure resection

**DOI:** 10.1038/s41598-021-99945-y

**Published:** 2021-10-18

**Authors:** Wataru Sato, Naotaka Usui, Reiko Sawada, Akihiko Kondo, Motomi Toichi, Yushi Inoue

**Affiliations:** 1 Psychological Process Team, Guardian Robot Project, RIKEN, 2-2-2 Hikaridai, Seika-cho, Soraku-gun, Kyoto, 619-0288 Japan; 2 National Epilepsy Center, NHO Shizuoka Institute of Epilepsy and Neurological Disorders, Urushiyama 886, Shizuoka, 420-8688 Japan; 3 Graduate School of Medicine, Kyoto University, 53 Shogoin-Kawaharacho, Sakyo, Kyoto, 606-8507 Japan

**Keywords:** Neuroscience, Cognitive neuroscience, Emotion, Social neuroscience

## Abstract

Detecting emotional facial expressions is an initial and indispensable component of face-to-face communication. Neuropsychological studies on the neural substrates of this process have shown that bilateral amygdala lesions impaired the detection of emotional facial expressions. However, the findings were inconsistent, possibly due to the limited number of patients examined. Furthermore, whether this processing is based on emotional or visual factors of facial expressions remains unknown. To investigate this issue, we tested a group of patients (*n* = 23) with unilateral resection of medial temporal lobe structures, including the amygdala, and compared their performance under resected- and intact-hemisphere stimulation conditions. The participants were asked to detect normal facial expressions of anger and happiness, and artificially created anti-expressions, among a crowd with neutral expressions. Reaction times for the detection of normal expressions versus anti-expressions were shorter when the target faces were presented to the visual field contralateral to the intact hemisphere (i.e., stimulation of the intact hemisphere; e.g., right visual field for patients with right hemispheric resection) compared with the visual field contralateral to the resected hemisphere (i.e., stimulation of the resected hemisphere). Our findings imply that the medial temporal lobe structures, including the amygdala, play an essential role in the detection of emotional facial expressions, according to the emotional significance of the expressions.

## Introduction

Detecting emotional facial expressions is an initial and indispensable component of conscious emotional communication^1^. Appropriate detection of others’ emotional expressions allows us to understand their emotional states, and thus regulates social behavior and promotes the creation and maintenance of social relationships^2^.

Experimental psychology studies of healthy participants have demonstrated that using the visual-search paradigm, emotional expressions were detected faster than emotionally neutral expressions^3–13^. For instance, Williams et al.^5^ instructed participants to search for target faces in arrays of distractor faces and tested the effects of emotional expressions on search behavior. The reaction times (RTs) for the detection of emotional (e.g., angry and happy) expressions among neutral expressions were shorter than those for the detection of neutral expressions among emotional expressions. Some studies suggested that this efficient detection of emotional expressions is due to the emotional, but not visual, factors of the expressions^7,8^. For instance, Sato and Yoshikawa^7^ instructed participants to search for normal emotional (angry and happy) facial expressions and their anti-expressions among neutral expression distractors. The anti-expressions were artificial facial expressions with visual changes quantitatively comparable with normal expressions and were categorized as emotionally neutral and rather natural^14^. The RTs for the detection of normal emotional expressions were shorter than those for the detection of anti-expressions. These data indicate that emotional facial expressions are efficiently detected because of their emotional significance.

A few previous neuropsychological studies have examined the neural substrates of this process, and found that a bilateral amygdala lesion impaired the detection of emotional facial expressions in visual-search tasks^15,16^. Specifically, Bach et al.^15^ tested two patients with bilateral amygdala damage, and a group of healthy controls, on a visual-search task in which participants searched for an angry target among an crowd of happy expressions or a happy target among a crowd of angry expressions. Although controls detected angry expressions more rapidly than happy ones, the patients showed the opposite pattern, detecting happy expressions more rapidly. Domínguez-Borràs et al.^16^ tested a patient with bilateral amygdala damage, and a control group, on a visual-search task in which participants searched for an emotional (fearful or happy) facial expression or a neutral facial expression among a crowd of neutral facial expressions. Whereas controls detected facial expressions of fear and happiness more rapidly than neutral expressions, the patient did not. Although the results are not completely consistent, collectively, these studies imply that amygdala lesions impair the detection of emotional facial expressions.

However, some issues regarding the involvement of the amygdala in the detection of emotional facial expressions remain unresolved. First, one study^17^ has reported no effect of amygdala lesions on the detection of facial expressions in a visual-search task. That study tested a bilateral amygdala-damaged patient and healthy controls using a visual-search task, in which participants detected a fear expression among a crowd with neutral expressions, or a neutral expression among a crowd with different neutral expressions. Both the patient and the controls detected fearful expressions more rapidly than neutral ones. These results imply that amygdala lesions may not impair the detection of emotional facial expressions. One plausible explanation for the inconsistent findings is the small sample size of the studies, which tested only one or two bilateral amygdala-damaged patients. Because such small samples do not provide reliable findings^18^, investigating this issue in a group of patients is warranted.

Furthermore, whether impaired detection of emotional expressions in amygdala-damaged patients is due to emotional or visual factors remains untested. Emotional and neutral facial expressions have not only different emotional significance but also different physical features (e.g., oblique eyebrows in angry expressions versus horizontal eyebrows in neutral expressions). Because some studies have demonstrated that several visual features, such as oblique lines and curves, were detected more efficiently than other features, such as horizontal lines^19,20^, it may be that the abnormal detection of emotional facial expressions in amygdala-damaged patients reported in previous studies reflected problems with visual processing. Regarding this issue, some functional neuroimaging studies have demonstrated that amygdala activity in response to emotional facial expressions reflected the emotional significance, but not the visual features, of the expressions^21,22^. Based on these data, we hypothesized that an amygdala lesion may impair the detection of emotional facial expressions, even after controlling for the visual elements of the expressions.

**Figure 1:**
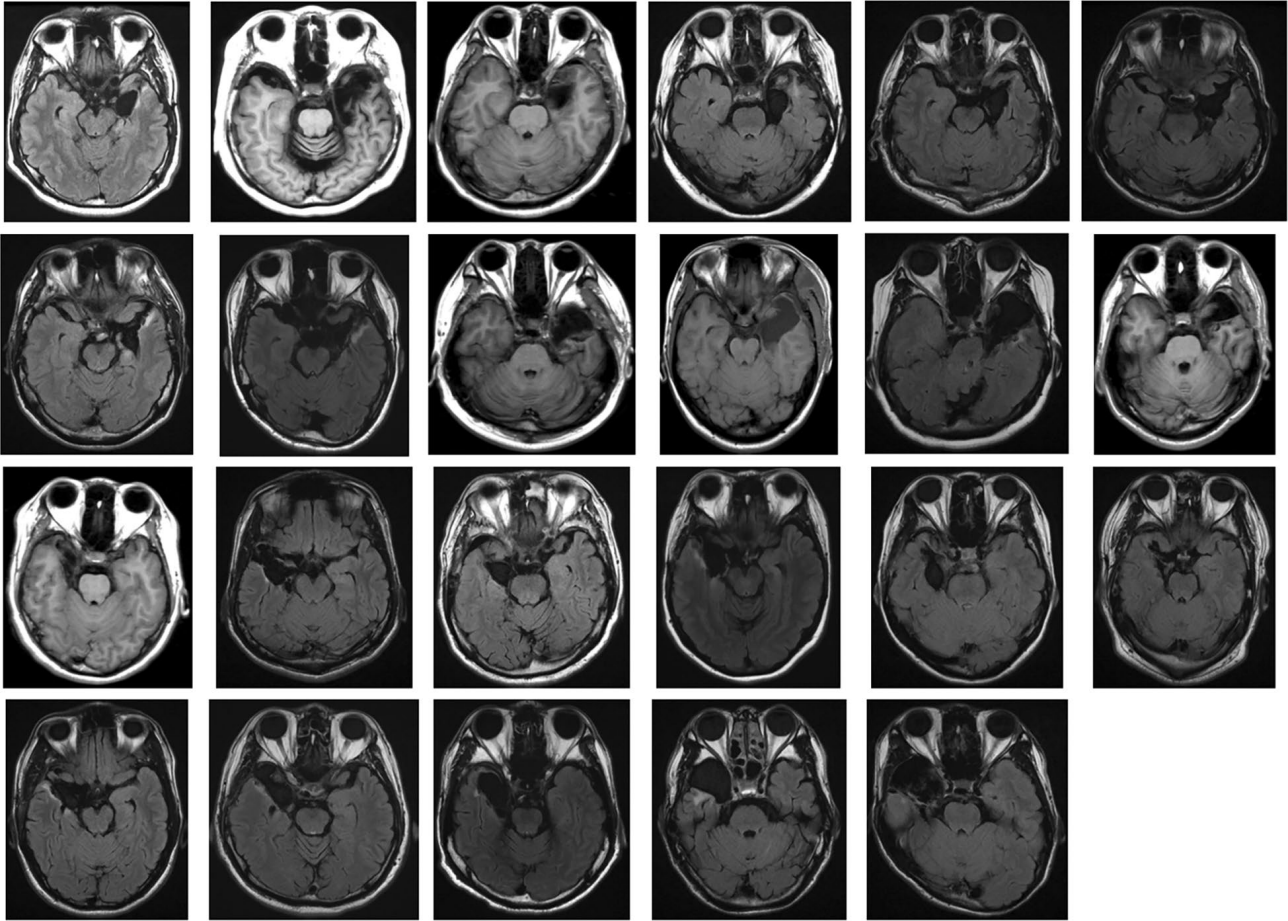
Anatomical magnetic resonance images of the temporal-lobe-resected patients. Left is shown on the right in the images.

**Figure 2:**
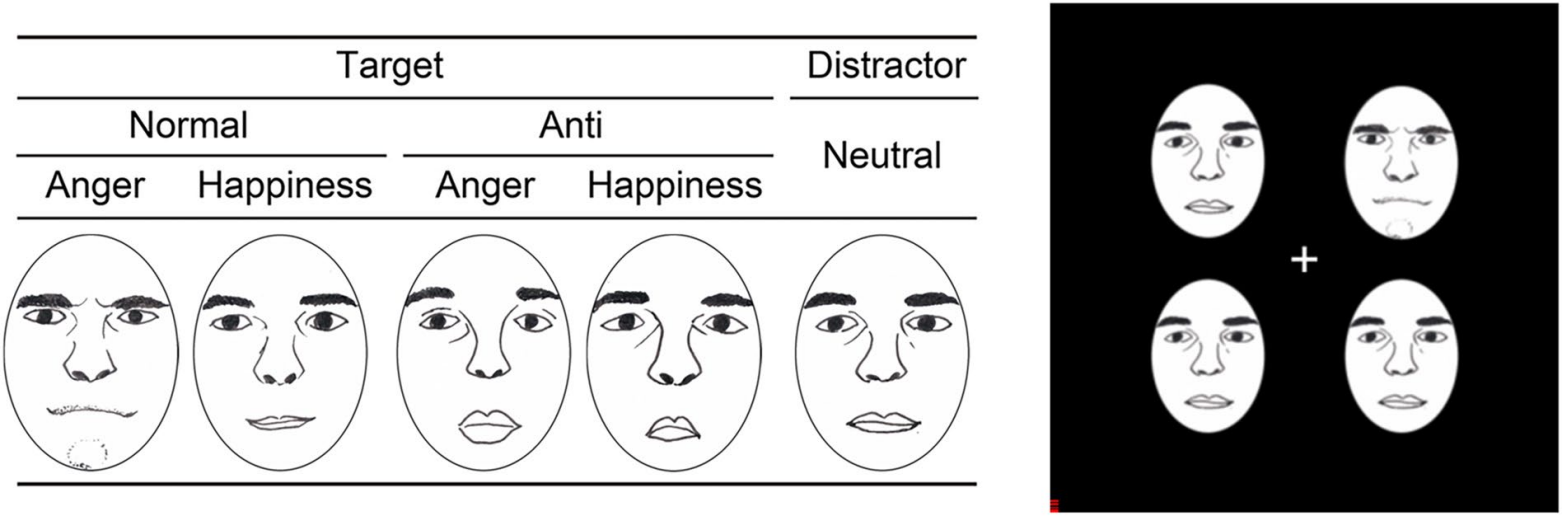
Illustrations of stimuli (left) and the visual search display (right). Actual stimuli were photographs of faces. Images in the figure are drawn by the author Prof. Wataru Sato.

To investigate this hypothesis, we tested a group of patients with unilaterally resected medial temporal lobe structures, including the amygdala (Fig. 1), using a visual-search paradigm. Normal facial expressions of anger and happiness in Caucasian models selected from a standard facial-expression database^23^, and their corresponding anti-expressions, were the target stimuli among a crowd with neutral expressions presented to the unilateral visual field (Fig. 2). Because the anti-expressions showed neutral emotions, but had visual feature changes equivalent to those between normal emotional and neutral expressions^14^, they allowed us to compare emotional and neutral facial expressions while controlling for the effects of basic visual processing. Because visual images presented in a unilateral visual field are primarily processed in the contralateral hemisphere^24^, we compared the RT required to detect normal expressions and anti-expressions between intact- and resected-hemisphere stimulation conditions. This visual half-field paradigm has been shown to effectively reveal the emotional and social processing profiles in one hemisphere of healthy participants^25–31^, split-brain patients^32–36^, and patients with unilateral medial temporal structure resection^37,38^. To confirm the emotional impact of normal expressions and anti-expressions, we also obtained subjective ratings of the stimuli from the patients, in terms of valence and arousal, and also investigated familiarity and naturalness as possible cognitive confounding factors^39^. Additionally, the performance of age-, sex-, and handedness-matched healthy controls was tested. We independently analyzed the data from the controls and compared their RTs for the detection of normal expressions versus anti-expressions between the left- and right-hemisphere stimulation conditions.

## Results

### Visual-search RT

**Figure 3:**
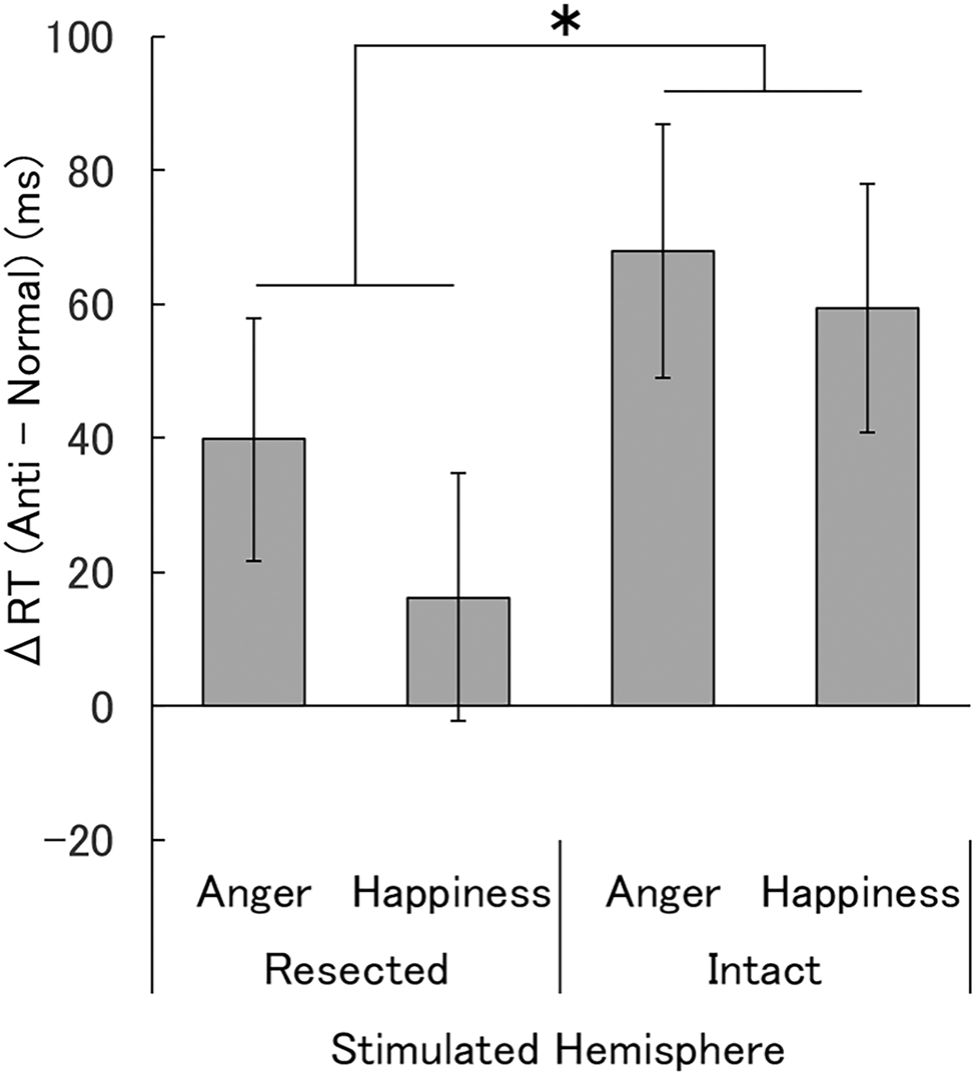
Mean (± *SE*) reaction time (RT) differences between the normal-expression and anti-expression conditions in temporal-lobe-resected patients. Asterisks indicate a significant simple main effect of stimulated hemisphere, indicating larger RT differences when the target faces were presented to the contralateral visual field (i.e., stimulation of the intact hemisphere) compared with the ipsilateral visual field (i.e., stimulation of the resected hemisphere). *p < 0.05.

The RTs of temporal lobe-resected patients obtained under each condition in the visual-search task are shown in Table 1, and RT differences between the normal-expression and anti-expression conditions are shown in Fig. 3. Two-way analysis of variance (ANOVA) for RT differences between normal expressions and anti-expressions, using stimulated hemisphere (resected, intact) and emotion (anger, happiness) as factors, showed a significant main effect of stimulated hemisphere, indicating faster detection of normal expressions versus anti-expressions when the intact hemisphere was stimulated (i.e., expressions were presented to the visual field contralateral to the intact hemisphere) than when the resected hemisphere was stimulated (i.e., expressions were presented to the visual field contralateral to the resected hemisphere) (*F*(1,22) = 5.22, *p* = 0.032, *η*^2^_*p*_ = 0.192). The main effect of emotion and the stimulated hemisphere × emotion interaction were not significant (*F*(1,22) < 0.73, *p* > 0.10, *η*^2^_p_ < 0.032). One-sample *t*-tests with Bonferroni correction for RT differences between normal expressions and anti-expressions revealed that the RT differences were significantly different from zero only under the intact-hemisphere condition with angry and happy expressions (*t*(22) = 3.59 and 3.19, Bonferroni-corrected *p* = 0.008 and 0.016, respectively).

**Table 1:** Mean (± *SE*) reaction times (ms) in the visual-search task in temporal-lobe-resected patients.

Stimulated hemisphere	Normal		Anti	
	Angry	Happy	Angry	Happy
Resected	883.3 (27.1)	928.0 (38.0)	923.1 (32.1)	944.2 (33.1)
Intact	866.2 (26.2)	918.3 (32.8)	934.1 (33.5)	977.7 (41.3)

To investigate the hemispheric functional asymmetry and resection method, four-way ANOVA was conducted for RT differences with additional between-subjects factors of resected side (left, right) and resection method (selective amygdala–hippocampus resection, temporal lobectomy). The results showed a significant main effect only of the stimulated hemisphere as in the above analysis (*F*(1,19) = 4.38, *p* = 0.043, *η*^2^_*p*_ = 0.199) and no other significant main effects or interactions (*F*(1,22) < 0.88, *p* > 0.10, *η*^2^_*p*_ < 0.089). The results imply no clear effect of hemispheric functional asymmetry or resection method.

**Figure 4:**
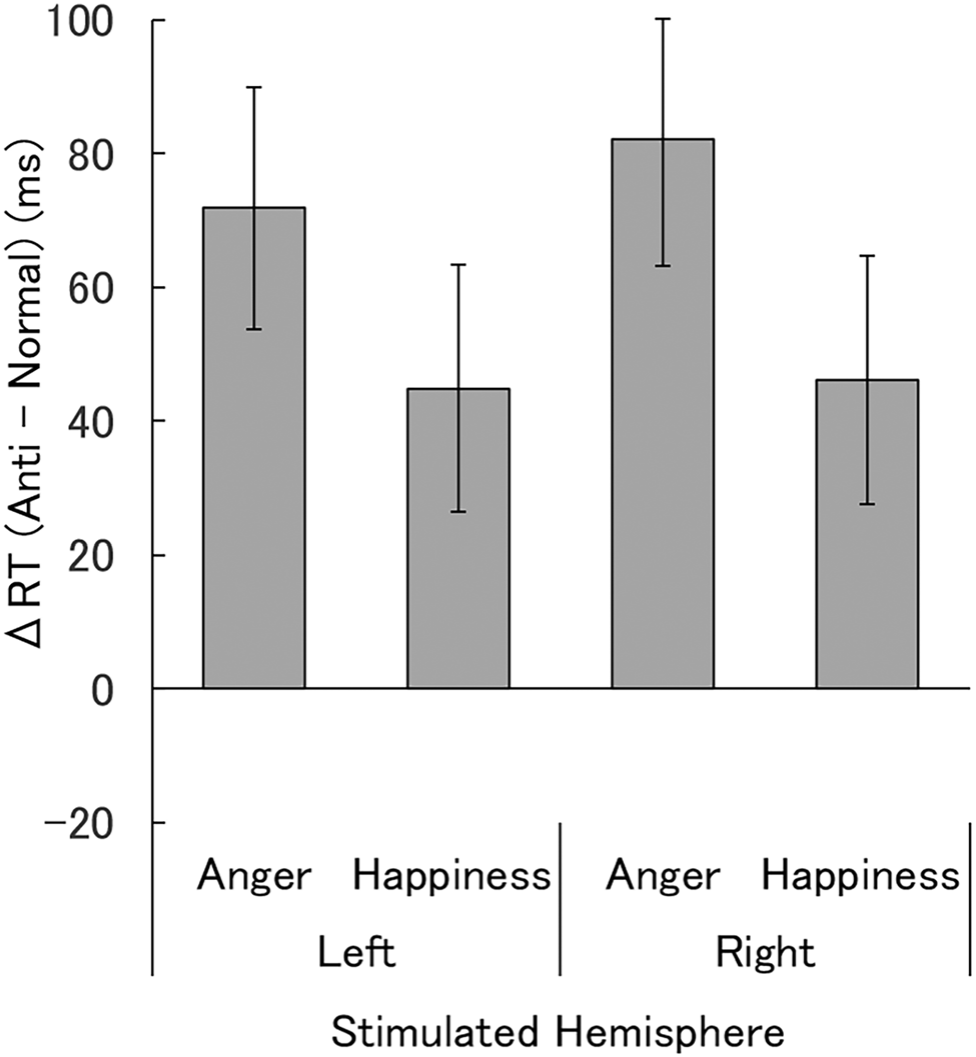
Mean (± *SE*) reaction time (RT) differences between the normal-expression and anti-expression conditions in controls.

The RT data from controls in the visual-search task are shown in Table 2 and Fig. 4. Two-way ANOVA for the RT differences between normal expressions and anti-expressions, with stimulated hemisphere (left, right) and emotion (anger, happiness) as factors, showed no significant main effect or interaction (*F*(1,22) < 2.33, *p* > 0.10, *η*^2^_p_ < 0.096). Bonferroni-corrected one-sample *t*-tests demonstrated that the RT differences between normal expressions and anti-expressions differed significantly from zero under all conditions of left and right hemispheric stimulation with angry and happy expressions (*t*(22) > 2.73, Bonferroni-corrected *p* < 0.049).

**Table 2:** Mean (± *SE*) reaction times (ms) in the visual-search task in controls.

Stimulated hemisphere	Normal		Anti	
	Angry	Happy	Angry	Happy
Left	940.6 (42.6)	953.2 (38.3)	1022.7 (40.9)	999.4 (42.1)
Right	933.7 (32.8)	951.9 (37.6)	1005.6 (32.1)	996.8 (35.9)

### Ratings

The subjective stimulus ratings of the temporal-lobe-resected patients are presented in Table 3. To assess the subjective emotional impact of the stimuli, valence and arousal ratings were evaluated. The familiarity and naturalness of the stimuli were included as possible covariates. The ratings were analyzed using two-way ANOVA with stimulus type (normal, anti) and emotion (anger, happiness) as factors.

**Table 3:** Mean (± *SE*) subjective ratings in temporal-lobe-resected patients.

Rating	Normal		Anti		Neutral
	Angry	Happy	Angry	Happy	
Valence	3.0 (0.4)	6.4 (0.4)	4.4 (0.3)	3.9 (0.3)	5.0 (0.3)
Arousal	5.0 (0.6)	5.4 (0.5)	4.3 (0.3)	4.7 (0.3)	4.5 (0.3)
Familiarity	3.6 (0.4)	6.7 (0.4)	4.3 (0.4)	3.8 (0.4)	5.6 (0.3)
Naturalness	4.0 (0.5)	6.3 (0.4)	5.3 (0.4)	4.6 (0.4)	6.1 (0.4)

For the valence ratings, the main effects of stimulus type (*F*(1,22) = 5.25, *p* = 0.032, *η*^2^_*p*_ = 0.193) and emotion (*F*(1,22) = 16.60, *p* = 0.001, *η*^2^_*p*_ = 0.430), as well as the interaction between stimulus type and emotion (*F*(1,22) = 22.47, *p* < 0.001, *η*^2^_*p*_ = 0.505) were significant. Follow-up analyses of the interaction showed significant simple main effects of stimulus type for both angry and happy expressions (*F*(1,44) > 8.64, *p* < 0.01), indicating more negative and positive valence ratings for normal angry and normal happy expressions, respectively, compared with their corresponding anti-expressions. For the arousal ratings, only the main effect of stimulus type was significant (*F*(1,22) = 4.38, *p* = 0.048, *η*^2^_*p*_ = 0.175), indicating higher arousal in response to normal expressions than to anti-expressions.

Analysis of the familiarity ratings revealed significant main effects of stimulus type (*F*(1,22) = 12.22, *p* = 0.002, *η*^2^_*p*_ = 0.357) and emotion (*F*(1,22) = 28.23, *p* < 0.001, *η*^2^_*p*_ = 0.562), and the significant interaction (*F*(1,22) = 34.31, *p* < 0.001, *η*^2^_*p*_ = 0.609). Follow-up analyses of the interaction showed that the simple main effect of stimulus type was significant only for happy expressions (*F*(1,44) = 44.20, *p* < 0.001); i.e., normal happy expressions were more familiar than anti-happy ones. For the naturalness ratings, the main effect of emotion (*F*(1,22) = 6.40, *p* = 0.019, *η*^2^_*p*_ = 0.225) and the stimulus type × emotion interaction (*F*(1,22) = 22.35, *p* < 0.001, *η*^2^_*p*_ = 0.504) were significant. Follow-up analyses of the interaction showed significant simple main effects of stimulus type on both anger and happiness (*F*(1,44) > 6.84, *p* < 0.05); i.e., naturalness ratings were higher for anti-angry than normal angry expressions, and for normal happy than anti-happy expressions.

The subjective ratings of the controls are shown in Table 4. The rating data from the controls were analyzed by two-way ANOVA using stimulus type and emotion as factors, like that described for the patients.

**Table 4:** Mean (± *SE*) subjective ratings in controls.

Rating	Normal		Anti		Neutral
	Angry	Happy	Angry	Happy	
Valence	2.9 (0.3)	6.9 (0.3)	4.4 (0.2)	4.4 (0.2)	5.4 (0.2)
Arousal	7.0 (0.2)	6.1 (0.3)	4.7 (0.2)	4.7 (0.3)	4.3 (0.3)
Familiarity	2.3 (0.2)	7.0 (0.2)	4.5 (0.3)	3.9 (0.2)	5.9 (0.3)
Naturalness	4.1 (0.5)	6.5 (0.3)	4.7 (0.4)	4.7 (0.3)	7.2 (0.3)

For the valence ratings, the main effect of emotion (*F*(1,22) = 81.77, *p* < 0.001, *η*^2^_*p*_ = 0.788) and the interaction between stimulus type and emotion (*F*(1,22) = 63.97, *p* < 0.001, *η*^2^_*p*_ = 0.744) were significant. Follow-up analyses of the interaction showed significant simple main effects of stimulus type for both angry and happy expressions (*F*(1,44) > 30.88, *p* < 0.001), indicating more negative and positive valence ratings for normal angry and normal happy expressions, respectively, compared with their corresponding anti-expressions. For the arousal ratings, only the main effect of stimulus type was significant (*F*(1,22) = 5.85, *p* = 0.024, *η*^2^_*p*_ = 0.210), indicating higher arousal in response to normal expressions than to anti-expressions.

For familiarity, the main effect of emotion (*F*(1,22) = 122.26, *p* < 0.001, *η*^2^_*p*_ = 0.847) and interaction (*F*(1,22) = 99.87, *p* < 0.001, *η*^2^_*p*_ = 0.819) were significant. Follow-up analyses of the interaction indicated significant simple main effects of stimulus type both on anger and happiness (*F*(1,44) > 40.10, *p* < 0.001), indicating that the familiarity ratings were higher for anti-angry expressions than for normal-angry expressions, and for normal-happy than for anti-happy expressions. For the naturalness ratings, the main effect of emotion (*F*(1,22) = 22.36, *p* < 0.001, *η*^2^_*p*_ = 0.504) and the stimulus type × emotion interaction (*F*(1,22) = 21.84, *p* < 0.001, *η*^2^_*p*_ = 0.498) were significant. Follow-up analyses of the interaction showed a significant simple main effect of stimulus type only for happy expressions (*F*(1,44) = 18.65, *p* < 0.001); i.e., the naturalness ratings were higher for normal happy than for anti-happy expressions.

## Discussion

Our results for the visual-search task in temporal-lobe-resected patients revealed that rapid detection of normal versus anti-expressions of anger and happiness was more evident with stimulation of the intact hemisphere than with stimulation of the resected hemisphere. Because we compared the RTs to normal expressions and their anti-expressions, which had featural changes quantitatively comparable with normal expressions, we controlled for visual factors. The subjective ratings of valence and arousal in the patients confirmed the difference in emotional impact between normal expressions and anti-expressions. Because a previous study suggested that cognitive aspects, such as familiarity, may modulate the detection of faces^39^, we also assessed the ratings of familiarity and naturalness. The results of these ratings were similar to those of previous studies of healthy participants^9–11^ and demonstrated different patterns from the detection performance and emotional ratings, implying that they did not account for the rapid detection of normal expressions versus anti-expressions. Visual-search results in healthy controls confirmed that normal angry and happy expressions were detected more rapidly than their anti-expressions in both hemispheres, thereby implying that both the right and left hemispheres can accomplish this process. The subjective rating results from controls also verified that normal expressions and anti-expressions had different emotional impact and that familiarity and naturalness ratings did not fit with the detection performance. The visual-search task results in the patients are consistent with those from previous studies showing that rapid detection of emotional expressions was impaired in patients with bilateral amygdala lesions^15,16^. However, inconsistent findings were reported with respect to the detection of emotional facial expressions by patients with bilateral amygdala lesions^17^, and no previous study has compared the effects of visual and emotional factors in this context. The results are also in line with previous findings from lesion studies showing that rapid, implicit emotional processing was impaired in patients with unilateral amygdala damage^37,40,41^. However, the detection performance of stimuli was not tested. To the best of our knowledge, this is the first study showing that the detection of facial expressions of anger and happiness is impaired by unilateral resection of medial temporal lobe structures, including the amygdala, in accordance with emotional, but not visual, aspects of the expressions.

Our findings suggest that the amygdala plays a crucial role in the detection of emotional facial expressions. This corroborates previous electrophysiological studies showing rapid activity in the amygdala during the processing of emotional facial expressions. For example, some intracranial field potential recordings in the amygdala showed that it was activated more rapidly in response to emotional expressions than to neutral expressions^42,43^. Another study indicated that the amygdala modulated activity in the visual cortex during the observation of facial stimuli^44^. Extending this literature, our findings imply that the amygdala is indispensable for the rapid processing of emotional facial expressions, in part due to its involvement in the conscious detection of facial expressions.

Several limitations of this study should be acknowledged. First, the resected region was not restricted to the amygdala; the anterior part of the hippocampus and parahippocampal gyrus were also resected. Several lesion and functional neuroimaging studies have shown that the human hippocampus, and adjacent structures, are involved mainly in spatial and episodic memory functions, implying a more important role of the amygdala in emotional processing^45,46^. However, animal anatomical studies revealed interconnections between the amygdala and hippocampal/parahippocampal regions^47^ and human neuroimaging studies showed functional coupling between these structures^48,49^, implying that the amygdala and hippocampal/parahippocampal regions act as a functional circuit. Future studies including patients with more amygdala-specific resection or damage are warranted to clarify the neural mechanisms underlying rapid detection of emotional facial expressions. Second, because we used normal expressions selected from a facial-expression database^23^ and their anti-expressions, the results may not be generalizable. Although this database^23^ has been used in a vast number of studies, the stimulus models are poorly diverse in terms of ethnicity^50^ and age^51^, and the database contains only acted facial expressions, which could differ from spontaneous emotional expressions^52,53^. Further investigation using different stimulus sets is needed to obtain robust findings. Third, although we created anti-expressions to make featural changes quantitatively comparable with those of the emotional facial expressions vis a vis the neutral expressions, we could not control for the holistic or configural aspects of facial expressions^54,55^. Some previous studies have suggested that holistic/configural aspects may be relevant to rapid detection of emotional facial expressions^56,57^. Future studies investigating the influence of these visual aspects may elucidate the impaired detection of facial expressions in patients with medial temporal lobe resection.

In conclusion, we tested a group of patients who had undergone unilateral medial temporal structure resection, including the amygdala, on a visual-search paradigm in which they detected normal facial expressions of anger and happiness and their anti-expressions among a crowd with neutral expressions. RTs to normal versus anti-expressions were shorter when the target face stimulated the intact hemisphere than when it stimulated the resected hemisphere. These findings imply that the medial temporal structures, including the amygdala, play an indispensable role in the detection of emotional facial expressions, in accordance with their emotional significance.

## Methods

### Participants

The patient group included 23 patients (8 females, 15 males; mean ± *SD* age = 32.7 ± 12.8 years) with medial temporal lobe structures that were unilaterally resected due to pharmacologically intractable seizures. Although three additional candidates were tested, their data were not analyzed because they displayed a visual deficit (*n* = 1; see “Procedure”), withdrew from the study (*n* = 1), or slept during the task (*n* = 1). We determined the sample size using an a priori power analysis. We used G*Power software^58^ (ver. 3.1.9.2) and assumed to contrast the intact- versus resected-hemisphere stimulation with an *α* level of 0.05, power of 0.80, and effect size *d* of 0.5 (strong). The results indicated that 21 participants would be required. All patients had undergone the surgical procedure more than 1 year before the experiment. Seizures were well controlled in most of the patients (*n* = 17, 3, 2, and 1 for Engel Classes^59^ I, II, III, and IV, respectively), and all were mentally stable during the experiments. Handedness was assessed using the Edinburgh Handedness Inventory^60^ (mean ± *SD* laterality quotient [LQ] = 76.2 ± 38.2); most patients were right-handed (i.e., LQ > 0; *n* = 22). Of the 23 participants, 12 (5 females, 7 males; mean ± *SD* age = 31.6 ± 12.1 years; mean ± *SD* LQ = 89.3 ± 12.3) and 11 (3 females, 8 males; mean ± *SD* age = 33.9 ± 14.0 years; mean ± *SD* LQ = 60.7 ± 50.8) had undergone resection in the left and right hemispheres, respectively. The resection method was selective amygdalohippocampectomy, which included the amygdala, anterior part of the hippocampus, and anterior parahippocampal gyrus, in 17 patients, and anterior temporal lobectomy, which included the amygdala, anterior part of the hippocampus, anterior temporal lobe neocortex (4–5 cm from the temporal pole), and anterior parahippocampal cortex, in six patients. Postsurgical magnetic resonance imaging confirmed resection of the target regions in all patients (Fig. 1). The healthy control group included 23 adults (8 females, 15 males; mean ± *SD* age = 28.0 ± 5.4 years; mean ± *SD* LQ = 84.1 ± 16.3). The control group was matched with the patient group for age (*t*(44) = 1.67, *p* = 0.115), sex (*χ*^2^(1) = 0.00, *p* = 1.000), and LQ (*t*(44) = 0.91, *p* = 0.369). All participants had normal or corrected-to-normal visual acuity, and all provided written informed consent following a full explanation of the procedure. This study was approved by the Ethics Committee of Shizuoka Institute of Epilepsy and Neurological Disorders, and was conducted according to institutional ethical provisions and the Declaration of Helsinki.

### Apparatus

The experiments were run on a Windows computer (HP Z200 SFF; Hewlett-Packard Company, Tokyo, Japan) with a 19-inch CRT monitor (HM903D-A; Iiyama, Tokyo, Japan) using Presentation 14.9 software (Neurobehavioral Systems, San Francisco, CA, USA). The resolution of the monitor was 1024 × 768 pixels, and the refresh rate was 100 Hz, as confirmed by a high-speed camera (1000 frames/s; EXILIM FH100; Casio, Tokyo, Japan). A response box with a 2–3-ms RT resolution was used to obtain responses (RB-530; Cedrus, San Pedro, CA, USA). A chin-and-forehead rest was used to maintain a distance of 0.57-m between the participant and the monitor.

### Stimuli

From a facial expression database^23^, we selected gray-scale photographs of a female (PF) and male (PE) model with angry, happy, and neutral expressions, with the teeth not showing. The models were not known to any of the participants. Anti-expressions were created from these normal expressions using morphing software (FUTON System; ATR, Soraku-gun, Japan). First, we manually identified the coordinates of 79 facial-feature points and readjusted them based on the coordinates of the iris of each eye. Next, the distances between each feature point of the emotional (angry and happy) and neutral facial expressions were calculated. Finally, anti-expressions were created by setting their feature positions to the same distance in the opposite direction. Two types of adjustments were made to the stimuli using Photoshop 5.0 (Adobe, San Jose, CA, USA). First, the photographs were cropped using an oval shape within the contour of the face, to eliminate factors irrelevant to the expression (e.g., hairstyle). Second, significant differences in contrast were eliminated, thereby removing possible identifying information. In addition, some minor color adjustments were made to a few pixels. The face stimuli were all 1.58° horizontally and 1.93° vertically. Photographs of normal expressions and anti-expressions of anger and happiness were used as target stimuli, and photographs of neutral expressions were used as distractor stimuli. The stimuli are illustrated in Fig. 2.

### Procedure

Each participant was tested individually. The experiment comprised three sessions for the patient group, i.e., visual field assessment, visual search, and rating sessions. For the control group, only the latter two sessions were conducted. Participants were instructed to keep their gaze on the fixation cross (0.86° × 0.86°) at the center of the display when the cross was presented throughout the sessions.

#### Visual field assessment

Participants were assessed for possible visual-field defects in four trials. In each trial, a white fixation cross (0.86° × 0.86°) was first presented in the center of the black display for 500 ms, followed by the target stimulus (white circle subtending 1.0°), which was presented for 200 ms in the corner of the square area where the faces were presented in the visual-search task. Participants were asked to look at the fixation cross, and then to point to the place where the target appeared. No participant included in the analysis showed any visual-field deficit.

#### Visual-search task

The visual-search task consisted of 512 trials presented in eight blocks of 64 trials, with an equal number of target-present and target-absent trials (i.e., 256). Targets were present in half of the trials as in the traditional visual-search task to prevent the effects of higher-level cognition and search strategies^61^. In the target-present trials, a target face was presented among three neutral faces, while the target-absent trials showed four neutral faces. Each target condition (normal anger, normal happiness, anti-anger, and anti-happiness for resected and intact hemisphere stimulation) was represented by 32 trials. The trial order was randomized across all conditions within a block. The interstimulus interval varied from 1300 to 1800 ms.

In each trial, after the fixation cross (0.86° × 0.86°) appeared for 500 ms in the center of the monitor, the 2 × 2 face stimulus array (4.30° × 4.30°) was presented against a black background until the participant responded. All faces were presented in the unilateral left or right visual field. An example of the stimulus display is shown in Fig. 2. Each facial array comprised pictures of a single model. Participants were instructed to look at a fixation cross, and then to decide whether one face was different, or all four faces were the same, by pushing predefined buttons on a response box using their left and right index fingers, as quickly and accurately as possible. The position of the response buttons was counterbalanced across participants.

#### Rating task

After the visual-search task, rating tasks for the target and distractor stimuli were performed. The stimuli were presented individually. Participants were asked to rate each stimulus in terms of emotional valence and arousal (i.e., the subjective ratings of the nature and intensity of the emotional experience), familiarity (i.e., the frequency with which they encountered the facial expressions depicted by the stimulus in daily life), and naturalness (i.e., the degree to which the expression depicted by the stimulus seemed natural) using a scale ranging from 1 to 9. The order of presentation of facial stimuli and rating items during the rating task was randomized.

### Data analysis

All statistical tests were performed using SPSS 16.0 J software (SPSS Japan, Tokyo, Japan). The *α*-level for all analyses was set to 0.05. For the RT analysis of patient data, mean RTs of correct responses in target trials were calculated for each condition and participant, with values ± 3 *SD* from the participant’s total mean excluded as artifacts. To simplify the analyses, RT difference scores were calculated for each participant by subtracting the RT for the normal expression condition from the RT for the anti-expression condition (positive values indicate faster reactions to normal expressions). The RT difference was then analyzed using two-way repeated-measures ANOVA with stimulated hemisphere (intact, resected) and emotion (anger, happiness) as factors. The RT differences were further tested for the difference from zero using one-sample *t*-tests (two-tailed) with Bonferroni correction; the alpha level was divided by the number of tests performed (i.e., 4). To investigate the effects of possible covariates, we also conducted ANOVAs of RT differences between normal-expression and anti-expression conditions, including the between-subjects factors of resected side (left, right) and resection method (selective amygdala–hippocampus resection, temporal lobectomy). Subjective ratings were also analyzed by ANOVA using the stimulus type (normal, anti) and emotion (anger, happiness) as factors. Preliminary analyses conducted for accuracy using two-way ANOVA with the same design as the above RT analysis showed no significant main effect or interaction (*F*(1, 22) < 1.02, *p* > 0.10, *η*^2^_*p*_ < 0.045). Hence, we report only the RT results, as in previous studies (e.g., [7]). Data from controls were analyzed in the same way, except that the factors included in the ANOVA for evaluation of RT differences were stimulated hemisphere (left, right) and emotion (anger, happiness).
